# Length of hospital stays and financial incentives: evidence from Dutch rehabilitation centers

**DOI:** 10.1007/s10198-023-01615-5

**Published:** 2023-08-09

**Authors:** Katalin Gaspar, Ramsis Croes, Misja Mikkers, Xander Koolman

**Affiliations:** 1 School of Business and Economics, Talma Institute / VU University Amsterdam, Section Health Economics, De Boelelaan 1085, 1081 HV Amsterdam, The Netherlands; 2 Dutch Healthcare Authority (NZa), Newtonlaan 1-41, 3584 BX Utrecht, The Netherlands; 3 Tilburg University, Warandelaan 2, 5037 AB Tilburg, The Netherlands

**Keywords:** Health care, Hospital, Hospital care, Financial incentives, I11, I13, I18

## Abstract

Non-linear reimbursement contracts in healthcare have been increasingly used to quantify providers’ responses to financial incentives. In the present research, we utilize a large one-off increase in the reimbursement of rehabilitation care to assess to what extent providers are willing to modify their treating behavior to maximize profits. In order to disincentivize the use of short inpatient stays for rehabilitation care, Dutch policy-makers have instated a two-part stepwise tariff-schedule. A lower tariff-schedule is applied for short hospital stays (≤ 14 days), while a higher tariff-schedule is utilized for longer treatments. Switching from one schedule to the other at day 15 of inpatient care leads to a sudden and large increase in tariffs. We show that, for most care-types, patients are seldom treated in an inpatient setting for less than 15 days, while the majority of patients are discharged after the threshold. Therefore, we conclude that the financial incentive at day 15 leads to considerable distortions in treatment. However, instead of discharging all patients at the threshold point where marginal tariffs are maximized, providers tend to continue treatment indicating altruistic behavior. As healthcare payment systems move away from piecewise reimbursement (e.g., fee-for-service arrangements), and services are increasingly ‘lumped’ together into e.g., DRGs and bundled payments, the likelihood of such discontinuities in tariff-schedules radically increases. Our research illustrates how such discontinuities in reimbursements can lead to distortions in the amount of healthcare provided contributing to the debate on optimal healthcare contracting design.

## Introduction

Recent health economics research has demonstrated that discontinuities in reimbursement design can lead to undesirable distortions and unnecessary oversupply of medical care. Economic theory suggests that a sudden increase in reimbursement based on treatment duration may induce providers to unnecessarily extend the length of treatment in order to receive higher tariffs, but once that tariff has been reached, the provider is incentivized to quickly discharge patients. This phenomenon is often referred to as strategic discharge behavior. Such behavior was confirmed by many recent papers: Medicare long-term acute care – Einav, Finkelstein, and Mahoney [3], Eliason et al. [4], mental healthcare—Douven, Remmerswaal, and Mosca [2], sepsis care—Bäuml and Kümpel [1], etc. Pletscher [11] in psychiatric care in Switzerland found that changes in marginal revenue per inpatient day can affect lengths of stay in the case of patients where the financial incentive was sufficiently large, while Pott et al. (2021) found no significant distortion in psychiatric care in Germany Pletscher [11], Pott et al. [12]. Whether or not such behavior is present is a pivotal question when designing optimal healthcare financing arrangements. As healthcare systems move away from piecewise (fee-for-service type) arrangements towards more bundling of care, it is inevitable that such discontinuities in reimbursements will be more prevalent. This could be along the lines of diagnosis-related groups (or DRGs), as in the case of Dutch rehabilitation care, where services are ‘lumped’ together into healthcare products based on diagnoses and treatment parameters, but it could also arise as result of a bundling strategy, where services are combined into clinically defined episodes of care Scott and Eminger [14]. It is, therefore, critical that healthcare policy-makers have a good understanding of whether or not such discontinuities lead to distortions in the provision of care.

**Fig. 1:**
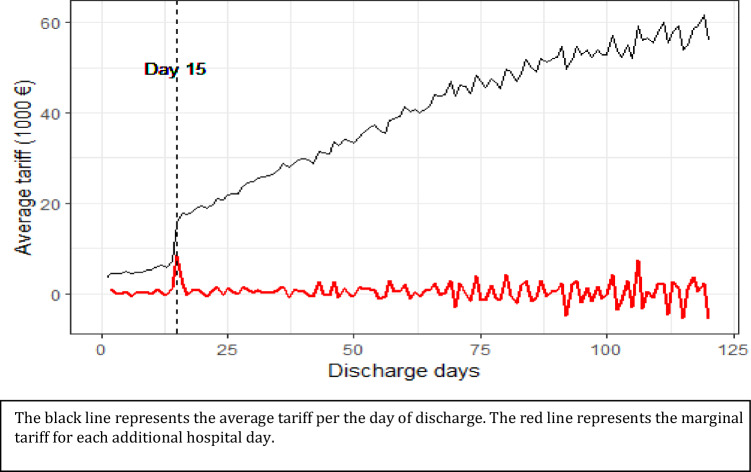
Brain disorders rehabilitation. Footnote: Own calculation for the years 2015–2018. Dashed black line represents day 15

In the present paper, we test this hypothesis in rehabilitation care in the Netherlands. In order to deter the use of short inpatient stays, Dutch policy-makers have devised a two-tier system for inpatient rehabilitation care. Hospital admissions up to 14 days (so-called non-clinical care) are financed using a lower tariff-schedule, while hospital admissions with 15 or more days of hospital stay (so-called clinical care) are reimbursed at a significantly higher tariff. The financial gains of extending treatments beyond 14 days is quite large. For example, in the case of brain disorders rehabilitation moving from 14 to 15 days of hospital stay, and thereby switching from non-clinical to a clinical tariff-schedule, can increase the provider’s reimbursement by approx. €9,000. This is the point where the marginal revenue per inpatient day is maximized, and where, assuming constant marginal costs throughout the hospital stay, the provider could maximize its profits.^1^ Following day 15 every additional day of treatment represents a relative loss to the provider in comparison to another patient discharged at day 15. On Fig. 1, we present the mean tariff per hospital discharge day (black line) and the marginal tariff for each additional inpatient day (red line) for brain disorder rehabilitation.^2^

In practice, only a small percentage of patients are discharged with inpatient days ranging between 1 and 14 days. Overall, 88.7% of patients admitted for hospital stay are treated for 15 or longer (see Table 1).

**Table 1:** Number of registered claims by care-type and length of stay

Condition	Length of stay	No. of Obs.	Perc.
Amputation	0 ≤ Days < 15	138	7.8
	Days ≥ 15	1633	92.2
Brain disorder	0 ≤ Days < 15	1616	9.4
	Days ≥ 15	15,547	90.6
Chronic pain	0 ≤ Days < 15	704	26.2
	Days ≥ 15	1988	73.8
Musculoskeletal systems	0 ≤ Days < 15	226	8.2
	Days ≥ 15	2528	91.8
Nervous system	0 ≤ Days < 15	273	10.8
	Days ≥ 15	2255	89.2
Organ Disorder	0 ≤ Days < 15	302	13.0
	Days ≥ 15	2018	87.0
Paraplegia	0 ≤ Days < 15	421	10.4
	Days ≥ 15	3628	89.6
Short rehabilitation	0 ≤ Days < 15	76	100.0
Overall	0 ≤ Days < 15	3756	11.3
	Days ≥ 15	29,597	88.7

Due to the long list of acute conditions that may require rehabilitation care (e.g., injuries and trauma, stroke, major surgeries, birth defects, developmental issues, or chronic pain) Krug and Cieza [8], Rauch, Negrini and Cieza [13] rehabilitation care tends to be an multi-disciplinary process. One that requires different care-givers (hospital medical specialists, physical therapists, speech therapists, occupational therapists and mental healthcare providers) to work closely together. Depending on the severity of the damage and the type of care required, rehabilitation care may be conducted in an inpatient or outpatient setting by general hospitals, university medical centers, independent treatment centers, rehabilitation centers and other multi-categorical providers.

In Gaspar and Koolman [5], we assess the prevalence of strategic discharges in outpatient rehabilitation facilities in response to a step-wise discontinuous reimbursement schedule in place based on treatment duration. In the outpatient setting, our findings suggest no response to incentives by traditional hospitals (general and academic hospitals), moderate response by rehabilitation centers and strong response by more profit-oriented independent treatment centers. In addition, we find that the financial health of the organization also plays a role in the magnitude of the response with higher probability of manipulation among providers in financial distress.

In the present paper, we turn our attention to a different type of discontinuity, one based on the number of hospital days. Moving from non-clinical tariffs (≤ 14 days) to clinical tariffs (> 15 days) can lead to a large (sometimes threefold) difference in tariffs, leading to a strong financial incentive for the provider to extend treatment up to this point and then to discharge the patient. As the focus of our paper is inpatient care and only rehabilitation centers and multi-categorical hospitals have facilities to admit patients with hospital stay, we limit our analysis to these two provider types.

Healthcare in the Netherlands is built on the fundamentals of regulated competition, a system in which residents are required by law to purchase basic health insurance from a selection of private insurers. These insurers are trusted with negotiating the price of care with individual providers. Although rehabilitation belongs to a special segment of care where maximum tariffs are set by the Dutch Healthcare Authority (NZa), providers may deviate downwards from this regulated price. In practice, variation in negotiated prices between providers and insurers remains minimal in this segment of care (see Appendix Table 3).

The insurance market consists of four major and a few smaller health insurers. Services provided by contracted providers are reimbursed fully above the yearly front-end deductible amount (between €350–385 during period of our study). In general, Dutch hospital staff either receive a monthly fixed salary or are paid on production-bases as self-employed. However, staff employed in rehabilitation care (including physicians, physiotherapists, occupational therapists, and so on) represent an exception and are almost exclusively paid on monthly fixed salaries. Therefore, the financial incentive inherent in the reimbursement scheme affects them indirectly, though the financial benefit of the healthcare organization in the form of potential for higher future wages and/or better working conditions.

### Data

The dataset used in this study was created by combining three national datasets: data from the Dutch risk adjustment system of the NZa, claims data of the Dutch national information system of health insurers (VEKTIS) and data on patient characteristics (VEKTIS Characteristics). In the present paper, we exclusively use *inpatient* claims registered by RCs and multi-categorical hospitals: 33,807 claims in total. Claims can be open for a maximum of 120 days, at which point they are automatically closed and declared.^3^ Rehabilitation care in the Netherlands is categorized into 7 types of care (amputation, brain disorder, chronic pain, musculoskeletal systems, nervous systems, organ disorders and paraplegia) and 1 additional categorized as short rehabilitation. Within inpatient care, about 4% of the care is categorized as short rehabilitation with a maximum weighted treatment hours (WHR) of 9 h. This period is devoted to establishing a diagnosis and setting up a treatment plan. A claim is registered as short rehabilitation if treatment is discontinued during this initial period. Once the treatment duration crosses the 9 weighted hour mark, a diagnosis and care-type is established and treatment begins. Even though care-type is not yet indicated for short rehabilitation claims, an initial diagnosis code is already registered. As eliminating these patients from our dataset would bias our analysis, we approximate the eventual care-type using the initial diagnosis code. This allows us to approximate the care-type the patient would follow had treatment continued.

## Methods

Our goal is to test whether the predicted probabilities of discharge differ week-by-week. In particular, we want to test whether the likelihood of a discharge significantly increases between week 2 (ending the day before the threshold) and week 3 (beginning the day of the threshold) after patient characteristics and fluctuations in week days are corrected for. This latter is important, as the day of the week a patient was admitted (and therefore due to be discharged) may unintentionally affect our results. (e.g., a patient that reaches the threshold day during the weekend is less likely to be discharged that day than someone who reaches the threshold day on a Friday).

We begin our analysis by expanding our dataset to include one line per claim per inpatient day and creating a binary variable indicating the discharge day. As a claim can be open for a maximum of 120 days, this will lead to 120 lines per claim. Next, we create dummy variables indicating 7 days of inpatient treatment in order to capture the change in the slope of the curve throughout the treatment process. The 7-day increments provide us with two advantages: firstly, it creates a natural separation between the inpatient day before the threshold (day 14) and the day of the threshold (day 15) and secondly, it represents one full week of treatment^4^. Hence, we end up with the following regression

$$Pr\left( {discharge_{it} = 1} \right) = \Phi \left[ {\mathop \sum \limits_{j = 1}^J week_j + \mathop \sum \limits_{j = 1}^J week_j *day_{it} + X_{it} + e_{it} } \right]$$

where a binary discharge variable indicates whether patient i is discharged on day t, a week variable indicates the number of weeks in hospital care ranging from 1 to 17, the day variable is an index indicating number of days in hospital care running from 1 to 120, and X refers to patient characteristics (sex, age and pharmaceutical spending in the previous year). The first term is meant to controls for overall changes in discharge probabilities for each week of treatment. The second term captures changes in discharge probabilities per inpatient day for each week. We use the above equation to calculate the predicted probability of discharge for each week and each inpatient day.

Next, we quantify the change in the predicted probability of moving from one week to the next by calculating the difference in probability between the last day of one week and the first day of the following week. This method allows us to identify any sudden changes in the likelihood of a discharge between weeks and also it allows us to account for any differences in patient characteristics and differences due to fluctuations in week-days. We present our main findings graphically.

## Results

### Section 1: Descriptive statistics

**Fig. 2:**
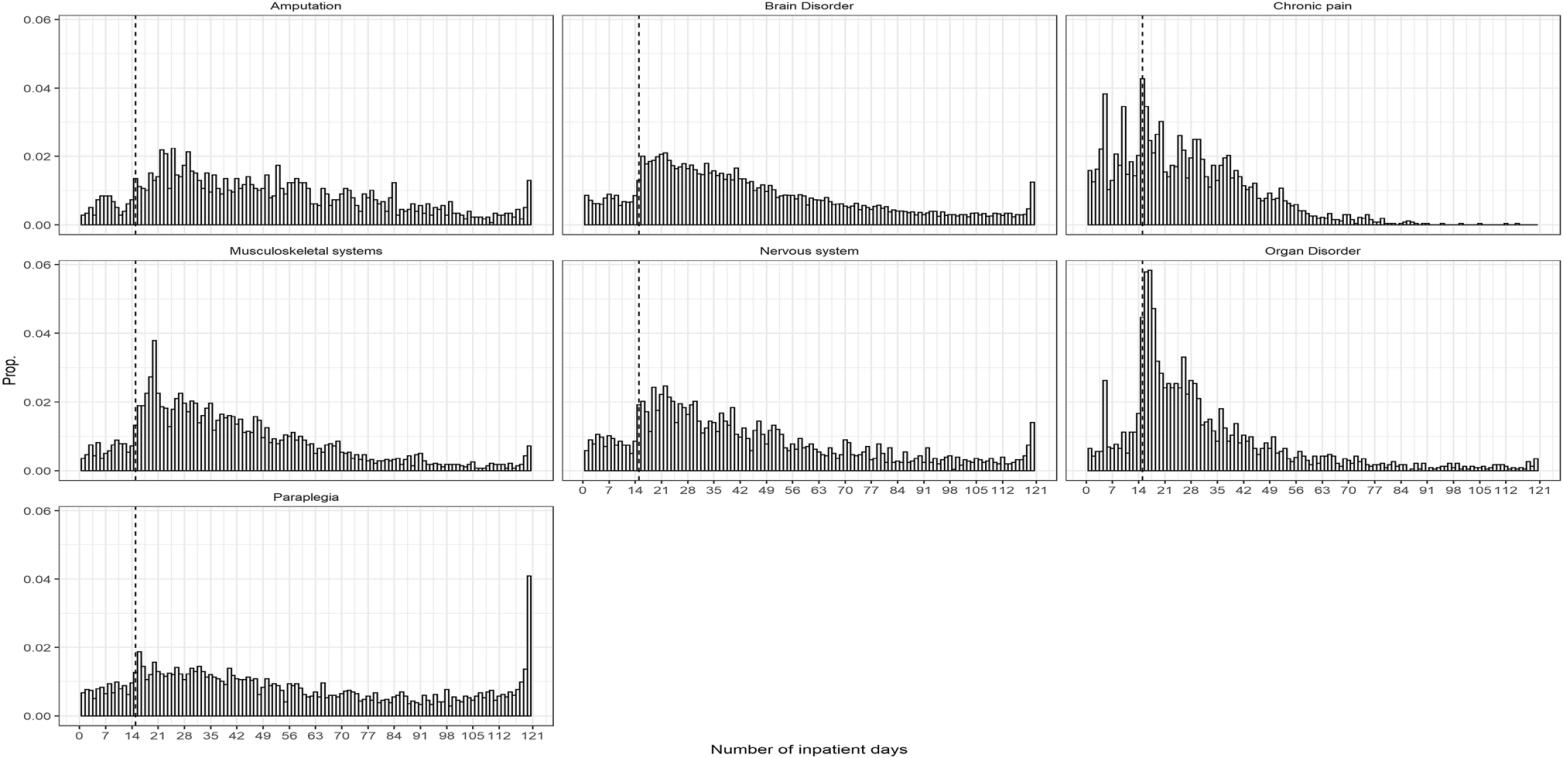
Distribution of inpatient days for all patients, 2015–2018. Footnote: Dashed black line represents day 15

In Table 1, we present the number of inpatient days for all years registered by rehabilitation centers and multicategorical hospitals. Although there is considerable variation between care-types, on average about 11.3% of the care falls within 1 and 14 days, while the rest (88.7%) involve 15 days or more days in hospital stay. We have a unique distribution with missing mass of registered claims from 1 up to 14 inpatient days for most care-types (brain disorder, musculoskeletal systems, nervous systems, organ disorder), and an excess mass up to approximately 20 days and a log-normal distribution beyond that point (See Fig. 2).

In Table 2, we present the descriptive statistics for our dataset. We use a variable indicating high pharmaceutical spending of the previous year to identify patients with previously high healthcare needs.^5^ This indicates a widely varying picture: while 70.9% of amputation patients and 66.7% of organ disorder patients consume costly pharmaceuticals, only 54% or less of patients do so in the rest of the sample. Similar variations in characteristics can be observed between care-types when looking at sex. While nearly 74% of patients in chronic pain are female, this figure is only 29% for amputation.

**Table 2:** Descriptive statistics

	Amputation	Brain disorder	Chronic pain	Musculoskeletal systems	Nervous system	Organ Disorder	Paraplegia	Short rehabilitation	Overall
	(*N* = 1787)	(*N* = 17318)	(*N* = 2700)	(*N* = 2782)	(*N* = 2546)	(*N* = 2328)	(*N* = 4133)	(*N* = 213)	(*N* = 33,807)
*High pharmaceutical consumption in the previous year*^a^									
Percentage	70.9	38.7	35.8	35.6	46.6	66.7	45.5	54.0	43.4
*Female*									
Percentage	29.0	38.5	73.8	44.5	50.0	38.7	34.7	44.1	41.8
*Age (in years)*									
Mean (SD)	61.0 (12.5)	56.9 (15.9)	34.5 (17.3)	45.8 (18.9)	51.5 (16.0)	55.3 (17.5)	55.8 (16.9)	55.4 (15.9)	53.8 (17.6)
*Weekdays*^b^									
Monday	312 (17.5%)	3421 (19.8%)	172 (6.4%)	502 (18.0%)	411 (16.1%)	586 (25.2%)	671 (16.2%)	18 (8.5%)	6093 (18.0%)
Tuesday	335 (18.7%)	3693 (21.3%)	446 (16.5%)	513 (18.4%)	550 (21.6%)	496 (21.3%)	832 (20.1%)	35 (16.4%)	6900 (20.4%)
Wednesday	376 (21.0%)	3620 (20.9%)	458 (17.0%)	609 (21.9%)	507 (19.9%)	498 (21.4%)	905 (21.9%)	43 (20.2%)	7016 (20.8%)
Thursday	398 (22.3%)	3620 (20.9%)	499 (18.5%)	618 (22.2%)	543 (21.3%)	408 (17.5%)	898 (21.7%)	47 (22.1%)	7031 (20.8%)
Friday	240 (13.4%)	2477 (14.3%)	603 (22.3%)	389 (14.0%)	357 (14.0%)	229 (9.8%)	521 (12.6%)	43 (20.2%)	4859 (14.4%)
Saturday	92 (5.1%)	341 (2.0%)	379 (14.0%)	101 (3.6%)	121 (4.8%)	79 (3.4%)	190 (4.6%)	19 (8.9%)	1322 (3.9%)
Sunday	34 (1.9%)	146 (0.8%)	143 (5.3%)	50 (1.8%)	57 (2.2%)	32 (1.4%)	116 (2.8%)	8 (3.8%)	586 (1.7%)

^a^Percentage of those with high pharmaceutical consumption in the previous year

^b^Number of patients discharged on each day of the week, in parenthesis as a percentage of the total

### Section 2: Calculating predicted probabilities after correction

**Fig. 3:**
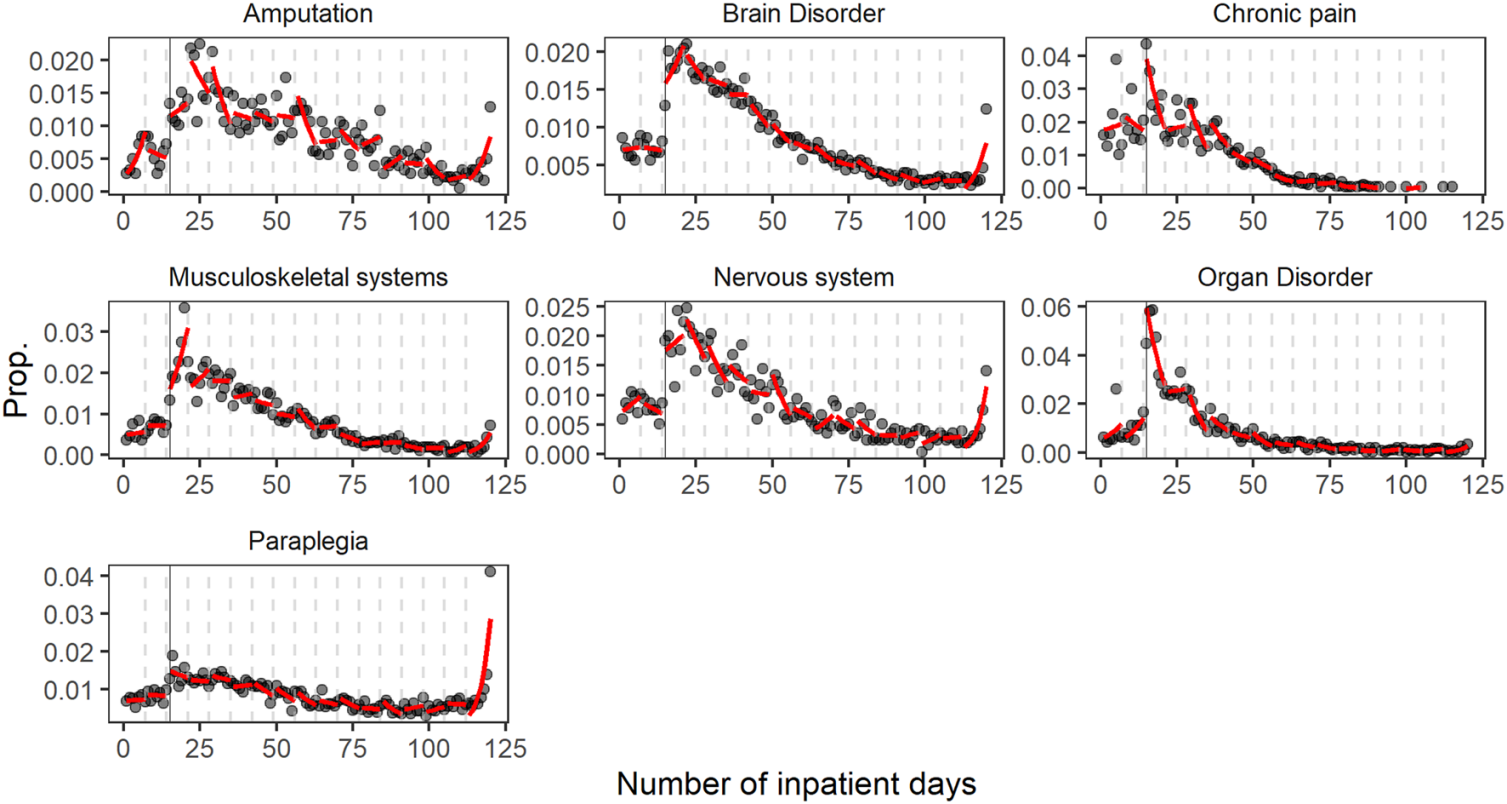
Predicted vs. actual probabilities of discharge per inpatient day, 2015–2020. Footnote 2: Black line represents day 15. Footnote 3: Dashed gray lines represent the beginning of each week

In the following section, we estimate the predicted probabilities of discharge for each day of inpatient care after correcting for patient characteristics and day of the week effects. In Fig. 3, we present the actual and predicted probabilities of discharge for each inpatient day. The graphical representation of predicted probabilities allows us to visually determine whether any sudden jumps occur in the predicted probability of a discharge within weeks. The string of weekly probabilities created using the week-dummies follows a relatively smooth curve for all care types, with the exception of a sudden jump between week 2 (ending one day before the threshold) and week 3 (beginning with the threshold) for the care-types of brain disorder, musculoskeletal systems, nervous systems, organ disorder, and weeks 2–5 for the care-type chronic pain and mental disorders (see full regression output in Appendix Table 3). The jump at day 15 is the most pronounced in the case of organ disorder. In addition, there is a sudden increase in discharges at (or near) 120 inpatient days for the majority of care-types. A larger share of patients is discharged at this point as this is the maximum number of inpatient days per claim allowed.

### Section 3: Partial differences

**Fig. 4:**
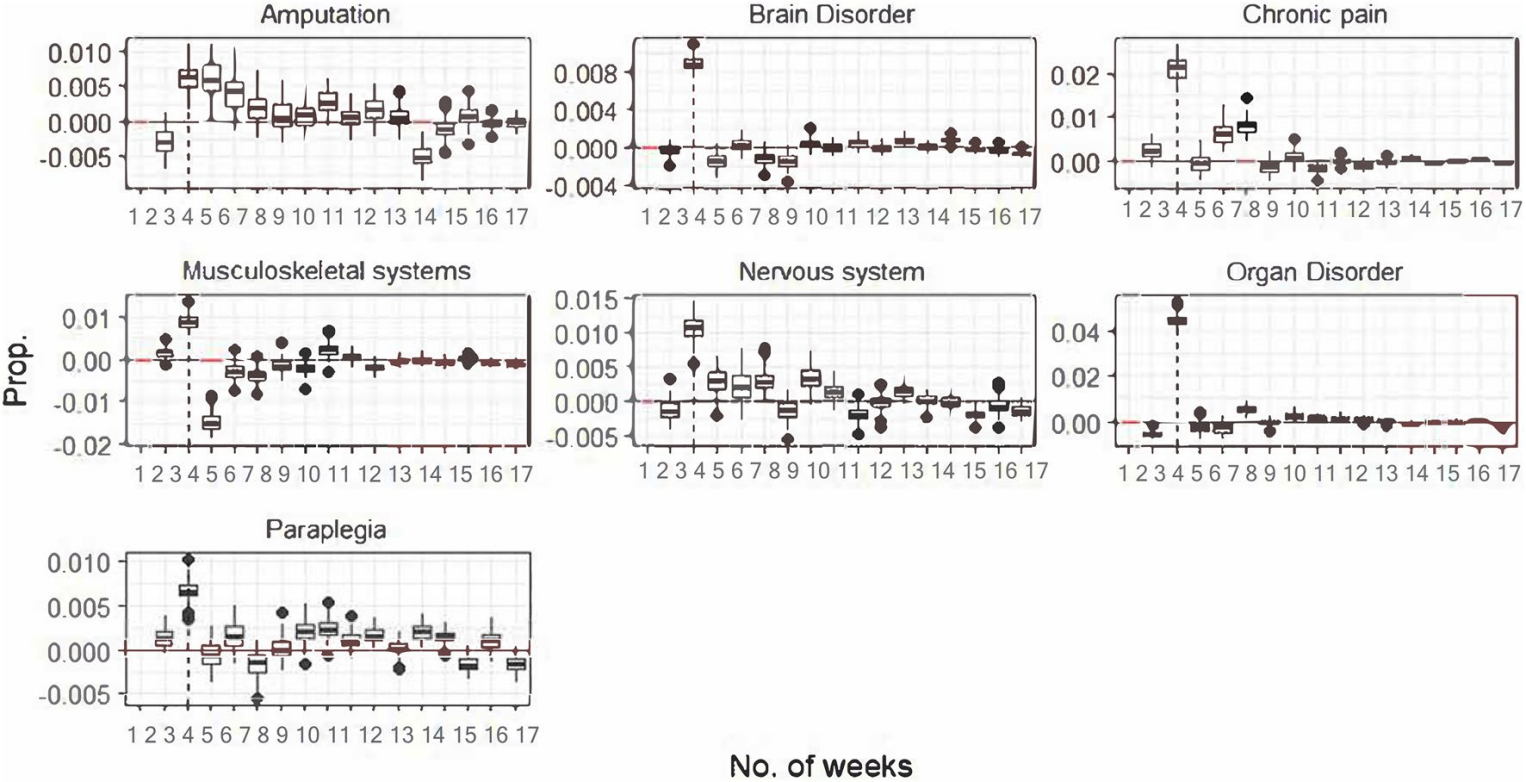
Bootstrapped partial differences

In the following section, we intend to capture the size of the gap between weeks by calculating the difference in the predicted probability of discharge between the last day of each week and the first day of the following week, which we refer to as weekly partial differences. Using the bootstrapping method, we create standard errors around these partial differences and test their statistical significance. As shown in Fig. 4, passrtial differences at week 3 are considerably larger than at any other week and they are statistically significant in all cases.

## Discussion

Earlier research has shown that healthcare providers respond to abrupt increases in reimbursement schedules by extending treatments to reach the higher tariff and then discharging patients directly following the threshold (e.g., Einav, Finkelstein, and Mahoney [3], Eliason et al. [4], while Pletscher [11] found that such distortions were only significant for patients where the financial incentive was sufficiently large and Pott et al. [12] found no significant distortion at all. The purpose of our study is to evaluate a similar financial incentive in the context of rehabilitation care in the Netherlands. Dutch rehabilitation care is reimbursed using a lower tariff-schedule for care provided either in an outpatient setting (with no inpatient days) or with up to 15 days of hospital stay. Once the patient has been in hospital care for 15 days or longer reimbursement increases to a higher, so-called clinical, tariff-schedule. The intention of the policy-maker in setting the 15-day rule was to deter rehabilitation providers from providing care with short hospital stays and to incentivize the use of outpatient care. Our results show that providers have responded strongly to the financial incentive, but instead of discharging all patients at day 15, as would be financially rational, providers tend to continue treatment beyond this point.

In Section 1, we descriptively present the distribution of claims per the number of inpatient days. Our results in Fig. 2 and Table 1 appear to correspond with the distortions caused by the abrupt introduction of the higher tariff. We find that patients are overwhelmingly discharged with 15 or more days of treatment, leading to a missing mass of registered claims between 1 and 14 days of inpatient care, followed by an excess of claims registered directly following the 15-day threshold (Fig. 1).

Although this unique distribution of hospital stays is in line with our hypothesis, it may be a result of the natural progression of treatments for patients with certain characteristics, severity of illnesses, or it may be influenced by the fact that the likelihood of a discharge fluctuates within the week. Therefore, in Section 2 we correct for patient characteristics (sex, age, and the level of previous pharmaceutical spending) and weekdays and run a logit regression estimating the probability of discharge for each inpatient day and adding dummy variables to indicate 7 day intervals. In Fig. 3, we present the distribution of actual and predicted probabilities of discharge for each care-type. Predicted probabilities per week follow an approximately smooth curve for most care-types, but a sudden jump can be observed between week 2 and 3, exactly where the 15-day threshold lies. This indicates that the partial difference in predicted probabilities at the 15-days remains significant after correcting for patient characteristics and weekday effects. This difference is significant for all care-types, but most pronounced for organ disorders.

Our findings show convincing evidence for strong providers’ response. It is probable that the threshold caused both over- and under-treatment, since certain patients, whose care would normally involve between 1 and 14 days of hospital stay, were denied appropriate inpatient care and treated entirely in an outpatient setting without appropriate 24/7 care. We also demonstrate that providers’ behavior did not follow a truly profit maximizing firm behavior, since this would have led to all patients discharged directly at day 15 where the marginal revenue per inpatient day was maximized. (See Fig. 1) Rather, we find that providers exhibited altruistic behavior, demonstrated by the distribution’s long right-hand tail. This is in contrast to the results found by Einav, Finkelstein, and Mahoney [3] and Eliason et al. [4] in U.S. long-term care hospitals, where providers discharged majority of patients directly following the threshold. But it is in line with findings of Pletscher [11] that found that marginal revenue can affect the length of stays, but only if the financial incentive is large enough.

Our results extend our findings in Gaspar-Koolman (2022) where a similar situation, albeit in monetary terms much smaller, incentive was analyzed in outpatient rehabilitation care in the Netherlands. In that study we found that rehabilitation centers showed only a moderate response to the financial incentive. Similarly to the findings of Pletscher [11], this leads us to conclude that the same institution may respond differently depending on the magnitude of the financial incentive.

In conclusion, our results lead to strong evidence for providers’ response to financial incentives created by the one-off increase in tariffs at 15 days in Dutch rehabilitation care. Our findings, therefore, support the notion that sudden changes in provider revenue can lead to distortions in the amount and quality of care provided. These findings seem to imply that moving away from volume-based reimbursement, especially one based on an easy-to-follow rule such as treatment duration, towards arrangements that are independent of volume, could minimize distortions and lead to overall more appropriate care. Payers could, for example, introduce global budgets, where providers are given a pre-specified and guaranteed budget to cover all costs associated with care for a fixed period of time (usually one year). These arrangements have been shown to incentivize cost-containment and to improve efficiency [6, 10, 16]. Another option could be to incentivise organizations based on quality of care and/or outcome. In rehabilitation care in practice this would mean that the provider has a financial incentive to intensively intervene at an early stage and for the period of time deemed appropriate for the best health outcomes. However, this could also lead to the cherry-picking of patients with the best prospective outcomes and, hence, the highest likelihood of generating the profits for the provider [9]. A mixture of arrangements, much like the alternative quality contracts in the United States, could be the most beneficial: a share of reimbursement amount could come from a guaranteed budget, while another share could be based on quality and health outcomes, thereby the incentives for strategic behavior are minimized [17].

## Data Availability

The data that support the findings of this study were used under license for the current study, and so are not publicly available.

## Appendix

See Appendix Tables 3 and 4.

**Table 3:** Ratio of regulated prices to negotiated prices per care-type, 2015–2018

Care-type	Type of care	Ratio Mean (%)	Ratio SD (%)
Amputation	Non-clinical	99.00	1.29
	Clinical	98.29	1.76
Brain Disorder	Non-clinical	98.53	2.04
	Clinical	98.20	1.95
Chronic pain	Non-clinical	98.68	2.05
	Clinical	98.82	1.99
Musculoskeletal systems	Non-clinical	98.36	1.92
	Clinical	98.17	1.63
Nervous system	Non-clinical	98.33	2.38
	Clinical	98.18	1.82
Organ disorder	Non-clinical	98.45	1.48
	Clinical	98.25	1.43
Paraplegia	Non-clinical	98.86	3.09
	Clinical	98.49	2.60

**Table 4:** Logit output

	Amputation	Brain disorder	Chronic pain	Musculoskeletal systems	Nervous system	Organ disorder	Paraplegia
(Intercept)	− 6.080 ***	− 4.958 ***	− 4.031 ***	− 5.309 ***	− 4.954 ***	− 5.177 ***	− 4.961 ***
	(1.061)	(0.586)	(1.018)	(0.749)	(0.454)	(0.748)	(1.018)
Week.dummies2	1.310 +	0.112	0.512	0.380	0.518	− 0.939 +	0.232
	(0.731)	(0.205)	(0.317)	(0.526)	(0.509)	(0.515)	(0.392)
Week.dummies3	1.209	0.124	2.838 ***	− 0.507	0.594	4.417 ***	1.072 *
	(0.804)	(0.211)	(0.421)	(0.505)	(0.535)	(0.408)	(0.478)
Week.dummies4	3.242 ***	1.766 ***	− 0.300	0.371	2.521 ***	1.266 +	0.711
	(0.915)	(0.281)	(0.696)	(0.705)	(0.704)	(0.655)	(0.687)
Week.dummies5	5.165 ***	1.130 **	4.054 ***	1.312	3.003 **	6.126 ***	1.163
	(1.251)	(0.374)	(0.895)	(0.895)	(0.999)	(1.048)	(0.848)
Week.dummies6	2.174	0.779	2.912 *	0.604	2.054	3.961 **	0.112
	(1.667)	(0.477)	(1.148)	(1.194)	(1.275)	(1.440)	(1.115)
Week.dummies7	2.595	2.516 ***	1.892	1.667	0.897	2.513	3.002 *
	(1.968)	(0.623)	(1.751)	(1.493)	(1.711)	(2.134)	(1.381)
Week.dummies8	1.994	2.626 **	2.636	1.396	6.685 **	9.249 **	3.799 *
	(2.253)	(0.801)	(2.279)	(1.955)	(2.047)	(2.947)	(1.710)
Week.dummies9	8.763 **	1.313	5.375	6.420 **	2.295	3.648	4.637 *
	(2.683)	(0.998)	(4.095)	(2.363)	(2.699)	(3.925)	(2.085)
Week.dummies10	0.706	3.328 **	− 4.549	− 0.412	− 4.973	6.356	1.642
	(3.434)	(1.200)	(5.262)	(2.916)	(3.452)	(4.605)	(2.475)
Week.dummies11	6.195	1.264	− 3.636	5.073	4.627	8.055	5.194 +
	(3.775)	(1.480)	(6.773)	(4.112)	(3.820)	(5.931)	(2.787)
Week.dummies12	− 0.879	4.597 **	22.321 +	− 2.298	7.390	− 1.188	0.792
	(4.151)	(1.704)	(13.569)	(5.388)	(4.633)	(7.665)	(3.422)
Week.dummies13	− 6.078	3.234	17.100	− 1.229	− 0.392	1.004	9.918 **
	(5.967)	(2.133)	(14.728)	(5.746)	(5.790)	(11.007)	(3.762)
Week.dummies14	1.522	3.527	− 5.803	7.451	− 4.804	− 13.709	− 3.488
	(6.486)	(2.379)	(47.512)	(7.869)	(6.223)	(11.489)	(3.903)
Week.dummies15	15.514 +	− 3.861	− 18.063	− 1.837	− 11.945	8.896	− 5.163
	(7.966)	(2.703)	(36.974)	(8.893)	(7.754)	(12.494)	(4.423)
Week.dummies16	− 5.127	− 1.411	− 1110.957	− 21.893 +	− 2.510	− 11.969	0.796
	(10.548)	(2.895)	(9515.268)	(11.245)	(7.652)	(12.083)	(4.123)
Week.dummies17	− 24.260 ***	− 24.326 ***	30.572	− 33.874 ***	− 38.708 ***	− 35.991 **	− 37.315 ***
	(6.905)	(2.227)	(58.185)	(7.764)	(6.104)	(11.965)	(3.011)
Days.index	0.198 **	0.008	0.016	0.020	0.050	0.107 *	0.006
	(0.064)	(0.017)	(0.027)	(0.049)	(0.041)	(0.043)	(0.035)
Week.dummies2 × days.index	− 0.233 **	− 0.016	− 0.053	− 0.020	− 0.088	0.028	− 0.010
	(0.087)	(0.024)	(0.038)	(0.065)	(0.059)	(0.059)	(0.047)
Week.dummies3 × days.index	− 0.171 *	0.039 +	− 0.150 ***	0.094 +	− 0.027	− 0.240 ***	− 0.027
	(0.075)	(0.020)	(0.035)	(0.055)	(0.049)	(0.047)	(0.043)
Week.dummies4 × days.index	− 0.247 ***	− 0.041 *	− 0.002	0.020	− 0.110 *	− 0.096 +	− 0.012
	(0.072)	(0.020)	(0.038)	(0.056)	(0.049)	(0.049)	(0.044)
Week.dummies5 × days.index	− 0.303 ***	− 0.017	− 0.143 ***	− 0.020	− 0.119 *	− 0.268 ***	− 0.023
	(0.074)	(0.020)	(0.039)	(0.056)	(0.051)	(0.054)	(0.044)
Week.dummies6 × days.index	− 0.212 **	− 0.010	− 0.094 *	− 0.007	− 0.085	− 0.190 ***	0.003
	(0.076)	(0.021)	(0.040)	(0.058)	(0.052)	(0.056)	(0.045)
Week.dummies7 × days.index	− 0.220 **	− 0.052 *	− 0.071	− 0.035	− 0.060	− 0.156 *	− 0.064
	(0.076)	(0.022)	(0.047)	(0.059)	(0.055)	(0.063)	(0.046)
Week.dummies8 × days.index	− 0.205 **	− 0.053 *	− 0.083	− 0.033	− 0.170 **	− 0.284 ***	− 0.074
	(0.076)	(0.023)	(0.051)	(0.062)	(0.056)	(0.070)	(0.048)
Week.dummies9 × days.index	− 0.319 ***	− 0.029	− 0.136 +	− 0.118 +	− 0.088	− 0.175 *	− 0.083 +
	(0.078)	(0.024)	(0.074)	(0.063)	(0.061)	(0.078)	(0.049)
Week.dummies10 × days.index	− 0.191 *	− 0.059 *	0.021	− 0.008	0.021	− 0.210 **	− 0.032
	(0.082)	(0.025)	(0.083)	(0.066)	(0.065)	(0.081)	(0.051)
Week.dummies11 × days.index	− 0.266 **	− 0.029	0.001	− 0.090	− 0.116 +	− 0.227 *	− 0.078
	(0.082)	(0.026)	(0.095)	(0.074)	(0.066)	(0.091)	(0.051)
Week.dummies12 × days.index	− 0.172 *	− 0.070 **	− 0.336 +	0.002	− 0.147 *	− 0.107	− 0.020
	(0.082)	(0.027)	(0.172)	(0.083)	(0.070)	(0.104)	(0.055)
Week.dummies13 × days.index	− 0.122	− 0.053 +	− 0.251	− 0.011	− 0.054	− 0.138	− 0.123 *
	(0.093)	(0.030)	(0.171)	(0.082)	(0.077)	(0.132)	(0.055)
Week.dummies14 × days.index	− 0.208 *	− 0.053 +	− 0.016	− 0.108	− 0.007	0.020	0.027
	(0.093)	(0.030)	(0.501)	(0.097)	(0.077)	(0.128)	(0.054)
Week.dummies15 × days.index	− 0.347 ***	0.021	0.111	− 0.012	0.057	− 0.211	0.041
	(0.101)	(0.031)	(0.362)	(0.100)	(0.086)	(0.130)	(0.056)
Week.dummies16 × days.index	− 0.152	− 0.003	9.869	0.169	− 0.035	− 0.011	− 0.015
	(0.116)	(0.031)	(84.958)	(0.114)	(0.081)	(0.118)	(0.051)
Week.dummies17 × days.index	0.015	0.196 ***	− 0.332	0.264 **	0.277 ***	0.188 +	0.317 ***
	(0.087)	(0.025)	(0.507)	(0.082)	(0.066)	(0.110)	(0.043)
Num.Obs	214,560	2,087,429	319,701	331,695	306,006	279,482	498,005
AIC	20,289.7	194,885.4	27,441.9	30,366.7	28,561.0	24,119.5	47,359.1
BIC	20,844.6	195,600.9	28,029.0	30,977.3	29,156.4	24,709.8	47,981.7
Log.Lik	− 10,090.833	− 97,385.716	− 13,665.958	− 15,126.344	− 14,224.500	− 12,003.768	− 23,623.529
F	8.082		22.981	24.147	16.796	42.539	14.168

**p* < 0.1; ***p* < 0.05; ****p* < 0.01

## Footnotes

1 This also assumes that there are enough new patients for every empty bed, i.e. there is (nearly) infinite inflow of new patients. This is assumption holds in the case of rehabilitation centers, also demonstrated by long waiting lists. [15].

2 In addition to the length of hospital stays, tariffs also increase with the weighted hours of treatment provided, which is equal to the hours of treatment times a multiplier used to adjust for variations in the cost of inputs. Therefore, payment is determined using two somewhat independent criteria: inpatient-days and weighted treatment hours. The number of inpatient days determines whether a non-clinical or clinical tariff-schedule is applicable, while weighted treatment hours determine *where* the patient’s care falls within a tariff-schedule. The fluctuation in average and marginal tariffs seen in Fig. 1 are caused by the differences in weighted treatment hours and variations in the negotiated tariff per provider.

3 Most patients are permanently discharged before or at 120 days, but a much smaller share remain in the hospital and another claim is opened. In this case, the same financial incentives apply as before: the provider is paid a lower (non-clinical) tariff for < 15 inpatient days and a higher tariff once the patient stays for 15 or more days. Unfortunately, the number of patients that have more than one claim consecutively is negligible (< 3%). Therefore, this group cannot be independently studied.

4 Under certain conditions, patients are encouraged to leave the treatment facility for the weekend and return back on Monday. These days are not counted as inpatient days (i.e. they do not count towards the 15 days) and therefore are excluded from our dataset.

5 This variable is borrowed from the Dutch risk assessment system of the NZa. The pharmaceutical costs groups (Farmaceutische kosten groepen or FKG) identifies high pharmaceutical spenders per chronic condition. In the present paper, we have combined all chronic groups into one variable, which is equal to 1 if the individual was a high pharmaceutical spender and zero otherwise [7].
